# Analysis of spelling errors from the ‘dyslexic sight words’ list

**DOI:** 10.3389/fpsyg.2024.1160247

**Published:** 2024-02-28

**Authors:** Luciana Cidrim, Andrea Oliveira Batista, Francisco Madeiro, Simone Aparecida Capellini

**Affiliations:** ^1^Catholic University of Pernambuco – UNICAP, Recife, Pernambuco, Brazil; ^2^São Paulo State University - UNESP. Marília, São Paulo, Brazil

**Keywords:** dyslexia, handwriting, teaching, learning, orthography

## Abstract

In this study the spelling errors of the ‘Dyslexic Sight Words - DSW’ list are analyzed according to the semiological classification. The spelling errors were made by schoolchildren with and without dyslexia. The high number of inaccuracies observed in the writing of the Group with dyslexia (GD) was often related to the complexity of syllabic structures and orthographic irregularity. The syllabic structures, in addition to the consonant-vowel (CV) pattern, often pose challenges for all students as they move through the alphabetic writing phase, early in literacy. This classification provides an understanding of the characteristics of Natural Spelling and Arbitrary Spelling, providing support for the teaching-learning of words by dyslexic students and is also relevant for the design of Portuguese language teacher training policies. In the teaching of the orthographic norm, the success and error when writing words should be followed by a reflection (metaorthographic skill) and monitoring of learning, both on the part of the teacher and on the part of the learner, reinforcing the knowledge of spelling patterns that will be triggered as the student is exposed to the explicit formal teaching of spelling.

## 1 Introduction

The establishment of orthographic writing is a complex learning process and takes several years (Justi et al., 2020) because, in the writing of words whose orthographies demand more than metaphonological skills, the mere exposure of the students to the written items (Cidrim et al., 2021) and the attempt to memorize orthographic rules are insufficient strategies to guarantee the reach of orthographic writing. This can be seen from research on orthographic processing (Apel et al., 2019) and the formal and explicit teaching of orthographic representations expected for the Portuguese language.

It can be inferred that spelling knowledge is correlated with orthographic lexical memory (Burt, 2006; Ehri, 2014; Apel, 2011, Apel et al., 2019; Batista and Capellini, 2020; Querido et al., 2021), which refers to the ability to use access to the spelling lexicon and phonological working memory in a combined way, for the correct writing of words. Orthographic lexical memory can be explained according to the cognitive model of orthography, regarding the planning and initial construction of the sentence or the written word, with the production of graphemes. This cognitive model features a processing unit called the grapheme production lexicon, lexical storage dedicated to retaining the spelling of familiar words stored in memory. In addition to this unit, another processing unit called the grapheme level has the primary function of being a short-term storage location (phonological working memory) responsible for maintaining the spelling of a word between retrieval and execution (Ellis, 1984; Ellis and Young, 1988).

In alphabetic writing systems, mastery of spelling depends on a solid understanding of phoneme-to-grapheme and spelling conventions. In addition, the learner needs to master the orthographic restrictions imposed by the orthographic depth of the language, which involves the complexity and unpredictability of phoneme–grapheme correspondences (Treiman and Bourassa, 2000; Schmalz et al., 2015). Orthographic depth varies along a continuum from shallow orthographies with simple and consistent phoneme–grapheme relations (e.g., Italian and Spanish) to deep orthographies with complex and inconsistent sound-letter mappings (e.g., English) (O’Brien et al., 2020). Brazilian Portuguese is more transparent in terms of decoding and opaque in terms of encoding (Santos and Navas, 2002; Cunha and Capellini, 2009).

With regard to the Portuguese language, the relationship between phonology and spelling produces less serious problems for word reading but is more pronounced for spelling (Caravolas, 2004). The explicit teaching of orthography is imperative based on the characteristics of orthographic transparency (Batista and Capellini, 2020).

In Brazilian Portuguese, which has an alphabetic writing system, the grapheme selection mechanism works based on two fundamental principles for orthographic notation: phonographic and semiographic. The conversion of graphemes into phonemes (letter-sound) and of phonemes into graphemes (sound-letter) refers to the phonographic principle, and these correspondences are more or less regular, depending on the reference to be analyzed, whether that of reading or writing (Mousinho and Correa, 2009). In the case of reading, the graphophonemic correspondence occurs more regularly (Cunha and Capellini, 2009); because of all the consonants of our alphabet, the letter <x > is the one that offers the greatest difficulty in decoding and can be read as /kiS/, /z/, /s/, and /ʃ/. In addition, regularity in reading also relies on phonological intuitions since, as Scliar-Cabral (2003) points out, the letters represent, better or worse, the phonemes. On the contrary, in writing, what appears is the occurrence of situations of irregularities in greater quantity, making spelling learning more costly (Scliar-Cabral, 2003). For Pinheiro (2006), what differentiates the processes of reading and writing is the direction of each process, from grapheme to phoneme in decoding and from phoneme to grapheme in encoding, directly influencing the way these skills are acquired, naturally, requiring different forms of teaching for each of them.

The conversions of graphemes into phonemes (letter-sound) and of phonemes into graphemes (sound-letter) refer to the semiographic principle, and these correspondences are, in a certain way, irregular, with the need to resort to grammar and, in particular, to morphology to get the correct spelling of a word. In essence, it refers to the correspondence between graphic symbols and units of meaning (Marec-Breton and Gombert, 2004).

While learning to write, the schoolchild has to deal with various levels of orthographic complexity (Gosse and Reybroeck, 2020). Regarding orthographic complexity, some words are easy to spell and are mastered early. As corresponding examples, one can cite the regular words (i.e., a high level of consistency of the correspondences between phonemes and graphemes). In the less transparent languages, some words will require more time to be mastered by children because they comprehend complex sounds and inconsistencies. Therefore, in the less transparent languages (e.g., English and French), the challenge is even greater for children because mastering the correspondences between phonemes and graphemes is not sufficient to reach correct spelling. Schoolchildren must also develop their lexical orthographic knowledge to be able to successfully write irregular words. Those challenges will be even greater in children with dyslexia (Gosse and Reybroeck, 2020). They struggle to learn the correspondences or to add new words to their orthographic lexicon in memory or both (Castles and Coltheart, 1993; Friedmann and Coltheart, 2018; Kohnen et al., 2018).

In alphabetic writing systems, accurate spellings rely on a solid knowledge of phoneme-to-grapheme and orthographic conventions (Treiman and Bourassa, 2000). Good writers need to be able to match speech sounds in a language (phonemes) with their accurate representation in written form (graphemes). Moreover, they need to master the orthographic constraints imposed by the orthographic depth of the language, which comprises the complexity and unpredictability of phoneme–grapheme correspondences (Schmalz et al., 2015). Orthographic depth varies along a continuum from shallow orthographies—with simple and consistent phoneme–grapheme relations—to deep orthographies—with complex and inconsistent sound-letter mappings (Katz and Frost, 1992). Concerning the orthographic depth of sound-to-print correspondences, European Portuguese, for example, has several phonemes with multiple representations (Lurdes de Castro, 2000), such as the phoneme /z/, which can be spelled [z], [s], or [x]. These multiple correspondences make the learning of spelling challenging, as reflected in the number of misspellings produced by beginning writers (Magalhães et al., 2020).

In the construction of the orthographic system by the student, characteristics of acquisition may vary in type and frequency, depending on age and grade (Nogueira and Cárnio, 2018). The difficulties are part of the process of appropriation of the orthographic system of the language, but they are overcome throughout schooling, starting with a more superficial knowledge of the sound-letter relations until the moment of being able to spell the irregularities of the written language (Sampaio et al., 2017; Chiaramonte and Capellini, 2019). In the case of students with specific learning disorders, such as dyslexia, there are difficulties that do not disappear with the progression of schooling (Cidrim and Madeiro, 2017; Kuerten et al., 2019).

Dyslexia results from differences in individual processing, often characterized by difficulties at the beginning of literacy, which compromises the acquisition of reading, writing, and spelling, in addition to presenting failures in cognitive, phonological, and/or visual processes (Reid, 2016). The identification of the specific cognitive and linguistic risk factors (e.g., poor phonological processing, low performance in rapid automatized naming, and weak verbal working memory) can help explain the weaknesses in reading and spelling development.

Among neurodevelopmental disorders, dyslexia impairs word reading accuracy, fluency, and comprehension (difficulty in understanding the meaning of what is read, e.g., one can read the text accurately but does not understand the sequence, relationships, inferences, or deeper meanings of what is read) (American Psychiatric Association, 2013; Sumner et al., 2014; Gosse and Reybroeck, 2020). Thus, given the deficits in cognitive–linguistic skills, phonological working memory, and phonographemic conversion, students diagnosed dyslexia, in many cases, also present with spelling disorders. The student who has the specific learning disorder with impairment in writing has deficits in spelling accuracy, grammar, and punctuation and damage to clarity and organization in written expression at different levels of severity (American Psychiatric Association, 2013).

In the Diagnostic and Statistical Manual of Mental Disorders – DSM-5 (American Psychiatric Association, 2013), there is neither the citation of the term dysorthography nor the elucidation of the types of spelling errors. It is worth highlighting the relevance of understanding spelling errors and their relationships with aspects inherent to the transparency or opacity of different writing systems. In its previous version, in DSM-4 (American Psychiatric Association, 1994), specific learning disorders with writing impairment were included in learning disorders with the nomenclature Disorder of Written Expression.

The occurrence of spelling disorders or dysorthographic manifestations with dyslexia or learning disorders is common since students in this situation have a deficient phonological system, causing changes in the letter-sound conversion and storage (Capellini et al., 2009; Batista et al., 2010; Apel et al., 2019; Chiaramonte and Capellini, 2022). This co-occurrence is frequent, as deficits in phonographemic conversion and linguistic knowledge, which are altered by dyslexia, also impair spelling learning since these mechanisms directly influence both diagnoses (Chiaramonte and Capellini, 2022).

A growing number of publications on spelling in the context of dyslexia have been observed (Mazur-Palandre, 2018; Gritz, 2020; Cidrim et al., 2021; Leonardi et al., 2021; Afonso et al., 2022; Bree et al., 2022; Bodard, 2022a,b; Bodard et al., 2023). Analyzing spelling errors can provide parameters to identify what is expected or not in learning the orthographic norm, in addition to assisting in interventions in the clinical and educational scopes. The results of the analysis of the types of persistent errors in the consolidation process of orthographic writing by students with dyslexia can be applied to assistive technologies in the clinical and educational context, as can already be observed in international publications (Dawson et al., 2019; Bäck et al., 2023) and recent national studies (Cidrim et al., 2018; Silva Neto et al., 2021) on the subject.

Manifestations of dyslexia vary among languages (Goulandris, 2003; Casani et al., 2022), subjects, and ages (Vellutino et al., 2004; Hasenäcker et al., 2020). This is also observed in studies with dyslexics in other languages, such as Italian (Casani et al., 2022), French (Joye et al., 2020; Bodard et al., 2023), Spanish (Rello et al., 2014; Afonso et al., 2022), German (Rauschenberger et al., 2016), and English (Bourassa and Treiman, 2003; Joye et al., 2020). For instance, the misspelling rate in dyslexic children is higher than in adults (Mazur-Palandre, 2018). However, experiments have evidence that adults with dyslexia have a continuing problem in the lexical domain, manifested in poor spelling ability (Afonso et al., 2015; Abadie and Bedoin, 2016).

In Brazil, word lists have been used to assess writing performance, more specifically, the spelling of students with learning difficulties (Batista et al., 2014; Arnaut et al., 2018), such as the ‘Dyslexic Sight Words – DSW’ list (Cidrim et al., 2021), composed of words often misspelled by schoolchildren with dyslexia. In the English language, ‘sight words’ are common words that students experience in written materials throughout their academic life (Grünke and Barwasser, 2019). From an early age, students are encouraged to learn how words should be written through lists of words called ‘sight words’ (McArthur et al., 2013, 2015). The ‘sight words’ lists are composed of words used with high frequency in the English language. Students are encouraged to memorize these words from an early age so that they can automatically recognize them without needing strategies to decode them (Ravitch, 2007). In addition, many of these words have an arbitrary spelling, and it is not possible for the student to recognize them just by decoding them. The words that make up the ‘sight words’ lists are divided into levels and introduced according to the frequency of appearance in the texts (Kear and Gladhart, 2003).

In this study, the classification based on the semiology of the errors was chosen because it addresses two general classes: the natural spelling (NS) errors and the arbitrary spelling (AS) errors. The aforementioned classification provides not only a descriptive character of the errors but also the evolutionary character, as it considers the order of orthographic acquisition in its nature, allowing the understanding of each type and the cognitive–linguistic factors involved.

Natural spelling has a direct relationship with language processing, especially with phonological and semantic skills, with the discovery of the alphabetic principle and with the acrophonic principle of the letters of the alphabet, characterizing the first phase of appropriation of writing, alphabetic writing, by beginning schoolchildren (Cervera-Mérida and Ygual-Fernández, 2006; Batista et al., 2014). Arbitrary spelling, both for rule-dependent and rule-independent spelling, is directly related to visual input memory, formation of a mental spelling lexicon, morphological and syntactic skills, and explicit knowledge of spelling rules, marking the third phase of appropriation of writing, orthographic writing, by more experienced students (Batista et al., 2014).

From natural spelling, the regular phoneme/grapheme correspondence (RPG) type of error refers to errors that affect the correspondence between phonemes and graphemes and are manifested as letter substitution. Omission or insertion of letters (OIL) happens when the student omits or adds a grapheme, changing the correct spelling of the word. In the case of the alteration in the syllabic structure (ASS) type of error, the semiological interpretation is that the errors may have been motivated by failures in the sequential processing or segmentation of syllables. In errors arising from the unconventional segmentation of word (USW), there is a change in the syllable structure of the words. Arbitrary Spelling errors implicitly bring an opacity in the phonographemic conversion. Thus, there is the type irregular phoneme/grapheme correspondence type 1 (IPG_1)—context-dependent orthographic rule that is related to the lack of knowledge and use of the positional rules of the graphemes representing the phonemes or even difficulty in analyzing the context of the position in the word where the phonemes will occupy to be represented by the graphemes. There is also the irregular phoneme/grapheme correspondence type 2 (IPG_2)—context-independent orthographic rule, in which total opacity is observed for phonographemic conversion and is related to the difficulty in storing the specific spelling of words when rules are absent, that can guide the orthographic notation (Cervera-Mérida and Ygual-Fernández, 2006).

A detailed approach to spelling errors, as it may vary across grades, can provide valuable information on the development of this skill (Magalhães et al., 2020; O’Brien et al., 2020; Afonso et al., 2022). These findings may improve our understanding of spelling development and provide useful hints to inform assessment and instructional spelling practices (Cervera-Mérida and Ygual-Fernández, 2006) since schoolchildren cannot overcome learning to read and write just by repeating and memorizing each word, although some spelling teaching approaches are still learning in the idea of repetition learning (Nunes and Bryant, 2014).

The present study aims to analyze in detail the spelling errors of the ‘Dyslexic Sight Words – DSW’ list according to the semiological classification made by Brazilian schoolchildren.

## 2 Materials and methods

### 2.1 Participants

This research uses the sample of the study conducted for the construction of the ‘Dyslexic Sight Words – DSW’ (Cidrim et al., 2021). Sixty Brazilian schoolchildren, aged between 8 years and 10 months and 12 years and 4 months, from 3rd to 6th grade of elementary school in private schools in the city of Recife/PE, wrote the ‘DSW’ list and were divided into group with dyslexia (**GD**) and group without dyslexia (**GWD**). **GD** was subdivided into **GD3**—10 children from the 3rd grade; **GD4**—eight children from the 4th grade; **GD5**—four children from the 5th grade; and **GD6**—eight children from the 6th grade; and **GWD** is subdivided into **GWD3**—10 children from the 3rd grade; **GWD4**—eight children from the 4th grade; **GWD5**—four children from the 5th grade; and **GWD6**—eight children from the 6th grade.

The group with dyslexia (GD) is composed of children with an interdisciplinary diagnosis of developmental dyslexia. All children were diagnosed by a team composed of at least three professionals: speech therapist, neuropsychologist, and neurologist or child psychiatrist.

The diagnosis followed DSM-5 criteria (American Psychiatric Association, 2013) confirmed through individually administered standardized performance measures and comprehensive clinical assessment: persistent difficulties in reading fluency, reading comprehension, written expression, and spelling; impaired academic skills and below expectations for the student’s chronological age; normal levels of intellectual functioning (intelligence quotient greater than approximately 70 [±5 points margin of measurement error]); and the use of the response to intervention model (RTI). Children diagnosed with other learning disorders, such as non-specific learning disorders, schoolchildren with low visual acuity, hearing and/or intellectual performance below the expected standards for their age, and schoolchildren with school difficulties (caused by unidentified factors) were excluded.

Participants in the group without dyslexia (GWD) are children without complaints of learning difficulties indicated by the Portuguese language teacher at the participants’ school. The pairing was conducted between the groups, and for this purpose, the same school year and gender were ensured, with the approximate age of the children.

The parents or guardians of the children signed the Free and Informed Consent Term, corresponding, in Portuguese, to Termo de Consentimento Livre e Esclarecido (TCLE), according to the resolution of the National Health Council CNS 196/96. Along with the TCLE, a term of assent was given to the participating students, TALE, given that the population involves children under 18 years of age. This term was written in accessible language and signed by the participant.

### 2.2 Materials

The ‘DSW’ list created by Cidrim et al. (2021) is made up of 60 words: *muito/very, quando/when, disse/said, também/also, vez/time, cachorro/dog, conseguiu/got, encontrou/found, gente/people, guerra/war, exemplo/example, ajuda/help, assim/so, brincar/play, futebol/soccer, menino/boy, animal/animal, carro/car, casa/house, então/then, homem/man, jeito/way, por isso/therefore, viajar/travel, a gente/us, almoço/lunch, assalto/assault, borracha/rubber, bruxa/witch, cabeça/head, caiu/fell, começou/started, de repente/suddenly, em cima/above, embaixo/under, enxergar/see, escola/school, faz/does, fazer/do, fez/did, girafa/giraffe, ninguém/nobody, pegue/take, porque/because, professora/teacher, quente/hot, tenho/have, alguém/somebody, amanhã/tomorrow, árvore/tree, certo/right, correr/run, exame/test, feliz/happy, presente/gift, relógio/clock, saudade/longing, tempo/time, tesoura/scissors, and galinha/chicken*. Spelling errors were classified by the Brazilian adaptation of the semiological classification of spelling errors (Batista et al., 2014) (Table 1).

**Table 1:** Brazilian adaptation of the semiological classification of spelling errors (Batista et al., 2014).

Spelling	Initials	Type designation	Examples
Natural	RPG	Regular Phoneme/Grapheme Correspondence	**Conseguiu** *(got)* – < *qu**a**nce**q**uio*>,**enxergar** *(see)* – < *en**j**e**i**ga*>,**de repente** (*suddleny*) – <***t****errepende*>
Natural	OIL	Omission or Insertion of Letters	**Conseguiu** *(got)* – < *cosegiu*>,**assalto** (*assault*) – < *a**l**caldo*>,**embaixo** (*under*) – <*ebaixo*>
Natural	ASS	Alteration in the Syllabic Structure	**Assalto** (*assault*) – < *a**l**çato*>, *<a**u**sato>*,***amanhã*** *(tomorrow)* – <*ama**han***>
Natural	USW	Unconventional Segmentation of Word	**Embaixo** (*under*) – < *em baixo*>,**por isso** (*therefore*) – <*porisso>*
Arbitrary	IPG_1	Irregular Phoneme/Grapheme Correspondence type 1 – Orthographic Rule Dependent of the context	**A gente** (*we*) – < *agent**i***>,**exemplo** (*example*) – < *e**z**enpo*>,**enxergar** (*see*) – < *e**m**xergar*>,**guerra** (*war*) – *<que**r**a>*
Arbitrary	IPG_2	Irregular Phoneme/Grapheme Correspondence type 2 – Orthographic Rule Independent of the context	**Exame** (*exam*) – < ***i****sami*>,**também** (*also*) – < *tabei*>,**começou** (*started*) – <*come**ss**o*u>
Arbitrary	AAW	Alteration in Accentuation of words	**Ninguém** (*nobody*) – < *ning**e**n*>,**árvore** (*tree*) – <***a****rvore*>

### 2.3 Procedures

The 30 children from the GD performed the dictation of 60 words in an individual session of up to 45 min in the private office of the first author of this research, and the 30 children from the GWD performed the dictation of 60 words, in groups, in a-30 min meeting at the school itself, in the presence of the first author of this research.

## 3 Data analysis

Statistical analysis was performed using the STATA/SE software, version 12.0, and Excel, version 2010. The tests used to verify the existence of an association were the chi-square test and the Fisher’s test. The chi-square test was not applied when the following condition failed: no more than 20% of cells can have an expected frequency lower than 5. In this case, the Fisher’s exact test is used.

The Kolmogorov–Smirnov normality test was used for quantitative variables. To evaluate the performance between the GD and GWD, the Mann–Whitney test was used (the data were not normally distributed). With the application of the Mann–Whitney test, it was possible to observe that there was a statistically significant difference in relation to the number of incorrect words written by the students from the GD and the GWD.

## 4 Results

The students from the GD wrote an average of 28.3 (47.16%) incorrect words, while GWD students wrote incorrectly an average of 4.3 words (7.16%) (Table 2).

**Table 2:** Average number of words written incorrectly by GD and GWD, minimum and maximum values, and *p*-value.

GROUP	Average ± SD	Median (Q1; Q3)	Minimum	Maximun	*p*-value
GD	28.3 ± 11.6	30.5 (17.5; 39.0)	10.0	47.0	<0.001*
GWD	4.3 ± 3.1	4.0 (1.0; 7.3)	0.0	9.0	

*Mann–Whitney Test.

Table 3 shows the number and percentage of students from the GD and GWD when writing words correctly and incorrectly and the value of *p*. It is observed that there was no statistically significant difference in many words, such as *muito (much), futebol (soccer), menino (boy), animal (animal), carro (car), casa (house), então (then), bruxa (witch), escola (school), girafa (giraffe), porque (because), tempo (time), and galinha (chicken)*, but in the general comparison between groups, there was a significant difference in the extent to which the GWD presented a higher percentage of correct answers.

**Table 3:** Number and percentage of students from the GD and GWD and the *p*-value.

Word	GD	GWD	*p*-value
	n (%)	n (%)	
1. Muito/much			
Correct	23 (76.7**%**)	29 (96.7**%**)	0.052*
Incorrect	7 (23.3**%**)	1 (3.3**%**)	
2. Quando/when			
Correct	24 (80.0**%**)	30 (100.0**%**)	0.024*
Incorrect	6 (20.0**%**)	0 (0.0)	
3. Disse/said			
Correct	13 (43.3**%**)	28 (933**%**)	<0.001**
Incorrect	17 (56.7**%**)	2 (6.7**%**)	
4. Também/also			
Correct	8 (26.7**%**)	25 (83.3**%**)	<0.001**
Incorrect	22 (73.3**%**)	5 (16.7**%**)	
5. Vez/time			
Correct	11 (36.7**%**)	30 (100.0**%**)	<0.001**
Incorrect	19 (63.3**%**)	0 (0.0)	
6. Cachorro/dog			
Correct	24 (80.0**%**)	30 (100.0**%**)	0.024*
Incorrect	6 (20.0**%**)	0 (0.0)	
7. Conseguiu/got			
Correct	2 (6.7**%**)	19 (63.3**%**)	<0.001**
Incorrect	28 (93.3**%**)	11 (36.7**%**)	
8. Encontrou/found			
Correct	19 (63.3**%**)	30 (100.0**%**)	<0.001**
Incorrect	11 (36.7**%**)	0 (0.0)	
9. Gente/people			
Correct	18 (60.0**%**)	30 (100.0**%**)	<0.001**
Incorrect	12 (40.0**%**)	0 (0.0)	
10. Guerra/war			
Correct	9 (30.0**%**)	29 (96.7**%**)	<0.001**
Incorrect	21 (70.0**%**)	1 (3.3**%**)	
11. Exemplo/example			
Correct	9 (30.0**%**)	28 (93.3**%**)	<0.001**
Incorrect	21 (70.0**%**)	2 (6.7**%**)	
12. Ajuda/help			
Correct	22 (73.3**%**)	30 (100.0**%**)	0.005*
Incorrect	8 (26.7**%**)	0 (0.0)	
13. Assim/so			
Correct	15 (50.0**%**)	29 (96.7**%**)	<0.001**
Incorrect	15 (50.0**%**)	1 (3.3**%**)	
14. Brincar/play			
Correct	17 (56.7**%**)	30 (100.0**%**)	<0.001**
Incorrect	13 (43.3**%**)	0 (0.0)	
15. Futebol/soccer			
Correct	21 (70.0**%**)	27 (90.0**%**)	0.053**
Incorrect	9 (30.0**%**)	3 (10.0**%**)	
16. Menino/boy			
Correct	29 (96.7**%**)	30 (100.0**%**)	1.000*
Incorrect	1 (3.3**%**)	0 (0.0)	
17. Animal/animal			
Correct	26 (86.7**%**)	30 (100.0**%**)	0.112*
Incorrect	4 (13.3**%**)	0 (0.0)	
18. Carro/car			
Correct	27 (90.0**%**)	30 (100.0**%**)	0.237*
Incorrect	3 (10.0**%**)	0 (0.0)	
19. Casa/house			
Correct	30 (100.0**%**)	30 (100.0**%**)	–
Incorrect	0 (0.0)	0 (0.0)	
20. Então/then			
Correct	24 (80.0**%**)	28 (93.3**%**)	0.254*
Incorrect	6 (20.0**%**)	2 (6.7**%**)	
21. Homem/man			
Correct	17 (56.7**%**)	29 (96.7**%**)	<0.001**
Incorrect	13 (43.3**%**)	1 (3.3**%**)	
22. Jeito/way			
Correct	12 (40.0**%**)	29 (96.7**%**)	<0.001**
Incorrect	18 (60.0**%**)	1 (3.3**%**)	
23. Por isso/therefore			
Correct	3 (10.0**%**)	24 (80.0**%**)	<0.001**
Incorrect	27 (90.0**%**)	6 (20.0**%**)	
24. Viajar/travel			
Correct	18 (60.0**%**)	29 (96.7**%**)	0.001**
Incorrect	12 (40.0**%**)	1 (3.3**%**)	
25. A gente/we			
Correct	2 (6.7**%**)	20 (66.7**%**)	<0.001**
Incorrect	28 (93.3**%**)	10 (33.3**%**)	
26. Almoço/lunch			
Correct	10 (33.3**%**)	28 (93.3**%**)	<0.001**
Incorrect	20 (66.7**%**)	2 (6.7**%**)	
27. Assalto/assault			
Correct	4 (13.3)	24 (80.0**%**)	<0.001**
Incorrect	26 (86.7)	6 (20.0**%**)	
28. Borracha/eraser			
Correct	14 (46.7**%**)	29 (96.7**%**)	<0.001**
Incorrect	16 (53.3**%**)	1 (3.3**%**)	
29. Bruxa/witch			
Correct	25 (83.3**%**)	30 (100.0**%**)	0.052*
Incorrect	5 (16.7**%**)	0 (0.0)	
30. Cabeça/head			
Correct	20 (66.7**%**)	30 (100.0**%**)	0.001**
Incorrect	10 (33.3**%**)	0 (0.0)	
31. Caiu/fell			
Correct	9 (30.0**%**)	30 (100.0**%**)	<0.001**
Incorrect	21 (70.0**%**)	0 (0.0)	
32. Começou/started			
Correct	10 (33.3**%**)	29 (96.7**%**)	<0.001**
Incorrect	20 (66.7**%**)	1 (3.3**%**)	
33. De repente/suddenly			
Correct	1 (3.3**%**)	21 (70.0**%**)	<0.001**
Incorrect	29 (96.7**%**)	9 (30.0**%**)	
34. Em cima/above			
Correct	8 (26.7**%**)	24 (80.0**%**)	<0.001**
Incorrect	22 (73.3**%**)	6 (20.0**%**)	
35. Embaixo/under			
Correct	7 (23.3**%**)	24 (80.0**%**)	<0.001**
Incorrect	23 (76.7**%**)	6 (20.0**%**)	
36. Enxergar/see			
Correct	9 (30.0**%**)	24 (80.0**%**)	<0.001**
Incorrect	21 (70.0**%**)	6 (20.0**%**)	
37. Escola/school			
Correct	30 (100.0**%**)	30 (100.0**%**)	–
Incorrect	0 (0.0)	0 (0.0)	
38. Faz/does			
Correct	8 (26.7**%**)	29 (96.7**%**)	<0.001**
Incorrect	22 (73.3**%**)	1 (3.3**%**)	
39. Fazer/do			
Correct	22 (73.3**%**)	30 (100.0**%**)	0.005*
Incorrect	8 (26.7)	0 (0.0)	
40. Fez/did			
Correct	7 (23.3**%**)	29 (96.7**%**)	<0.001**
Incorrect	23 (76.7**%**)	1 (3.3**%**)	
41. Girafa/giraffe			
Correct	26 (86.7**%**)	30 (100.0**%**)	0.112*
Incorrect	4 (13.3**%**)	0 (0.0)	
42. Ninguém/nobody			
Correct	3 (10.0)	20 (66.7)	<0.001**
Incorrect	27 (90.0)	10 (33.3)	
43. Pegue/take			
Correct	14 (46.7**%**)	29 (96.7**%**)	<0.001**
Incorrect	16 (53.3**%**)	1 (3.3**%**)	
44. Porque/because			
Correct	27 (90.0**%**)	30 (100.0**%**)	0.237**
Incorrect	3 (10.0**%**)	0 (0.0)	
45. Professora/teacher			
Correct	20 (66.7**%**)	30 (100.0**%**)	0.001**
Incorrect	10 (33.3**%**)	0 (0.0)	
46. Quente/hot			
Correct	19 (63.3**%**)	29 (96.7**%**)	0.001**
Incorrect	11 (36.7**%**)	1 (3.3**%**)	
47. Tenho/have			
Correct	22 (73.3**%**)	29 (96.7**%**)	0.026*
Incorrect	8 (26.7**%**)	1 (3.3**%**)	
48. Alguém/somebody			
Correct	4 (13.3**%**)	25 (83.3**%**)	<0.001**
Incorrect	26 (86.7**%**)	5 (16.7**%**)	
49. Amanhã/tomorrow			
Correct	15 (50.0**%**)	27 (90.0**%**)	0.001**
Incorrect	15 (50.0**%**)	3 (10.0**%**)	
50. Árvore/tree			
Correct	13 (43.3**%**)	28 (93.3**%**)	<0.001**
Incorrect	17 (56.7**%**)	2 (6.7**%**)	
51. Certo/right			
Correct	18 (60.0**%**)	30 (100.0**%**)	<0.001**
Incorrect	12 (40.0**%**)	0 (0,0)	
52. Correr/run			
Correct	16 (53.3**%**)	27 (90.0**%**)	0.002**
Incorrect	14 (46.7**%**)	3 (10.0**%**)	
53. Exame/exam			
Correct	6 (20.0**%**)	26 (86.7**%**)	<0.001**
Incorrect	24 (80.0**%**)	4 (13.3**%**)	
54. Feliz/happy			
Correct	21 (70.0**%**)	30 (100.0**%**)	0.002*
Incorrect	9 (30.0**%**)	0 (0.0)	
55. Presente/gift			
Correct	24 (80.0**%**)	30 (100.0**%**)	0.024*
Incorrect	6 (20.0**%**)	0 (0.0)	
56. Relógio/clock			
Correct	11 (36.7**%**)	24 (80.0**%**)	0.001**
Incorrect	19 (63.3**%**)	6 (20.0**%**)	
57. Saudade/longing			
Correct	14 (46.7**%**)	27 (90.0**%**)	<0.001**
Incorrect	16 (53.3**%**)	3 (10.0**%**)	
58. Tempo/time			
Correct	22 (73.3**%**)	27 (90.0**%**)	0.095**
Incorrect	8 (26.7**%**)	3 (10.0**%**)	
59. Tesoura/scissors			
Correct	22 (73.3**%**)	30 (100.0**%**)	0.005*
Incorrect	8 (26.7**%**)	0 (0.0)	
60. Galinha/chicken			
Correct	27 (90.0**%**)	30 (100.0**%**)	0.237**
Incorrect	3 (10.0**%**)	0 (0.0)	

*Fisher’s exact Test.

**Chi-square Test.

**Figure 1:**
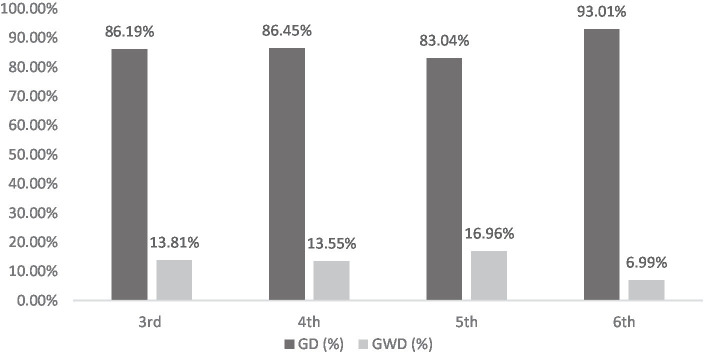
Percentage of words written incorrectly by school grade by GD and GWD.

Figure 1 presents the percentage of words written incorrectly by GD and GWD organized by school grade. A total of 387 incorrectly written words were observed in GD3, consisting of 10 students, corresponding to 387:10 = 38.7 wrong words by students on average. In short, of the 60 words that make up the dictation, each GD3 student wrote approximately 39 words incorrectly. In GWD3, 62 incorrectly written words were observed, corresponding to 62:10 = 6.2 incorrect words by students on average. In short, of the 60 words that make up the dictation, each GWD3 student miswrote around six words. In percentage terms, 387/_(387 + 62)_ = 86.19% of all misspelled words in the 3rd grade were committed by GD3 students and 62/_(62 + 387)_ = 13.81% of all misspelled words were committed by GWD3 students.

The GD4 is made up of 8 students. In this group, a total of 236 incorrectly written words were observed, which leads to an average number of incorrect words by students corresponding to 236:8 = 29.5. This means that, on average, out of the 60 words in the dictation, each GD4 student got around 30 words wrong. The GWD4, in turn, also made up of 8 students, recorded a total of 37 incorrectly written words, which leads to an average of 37:8 = 4.6 incorrect words by GWD4 students. In short, of the 60 words in the dictation, each GWD4 student wrote around 5 words wrongly on average. From the point of view of percentage values for the 4th grade, it was observed that 236/_(236 + 37)_ = 86.45% of incorrectly written words were written by GD4 students. Thus, 13.55% of words written incorrectly in the 4th grade are due to students in GWD4.

In the case of GD5, with 4 students, 93 incorrectly written words were observed, which leads to an average of 93:4 = 23.2 incorrect words by the student. In short, of the 60 words in the dictation, each GD5 student got around 23 words wrong. For GWD5, there were a total of 19 incorrect words, which corresponds to 19:4 = 4.8 incorrect words by the student. In percentage terms, within the 5th grade, 93/_(93 + 19)_ = 83.04% of the wrong words were written by GD5 students, while 16.96% of incorrectly written words were written by GWD5 students.

For GD6 with 8 students, 133 words were written incorrectly, which leads to 133:8 = 16.6 incorrect words by the student. This means that each student gets around 17 of the 60 words in the dictation wrong. In the case of GWD6, the number of incorrect words by the student is 10:8 = 1.3 words. From the point of view of percentage values, we have that 133/_(133 + 10)_ = 93.01% of all incorrect words in the 6th grade were committed by GD6 students and 10/_(133 + 10)_ = 6.99% were made by GWD6 students.

**Figure 2:**
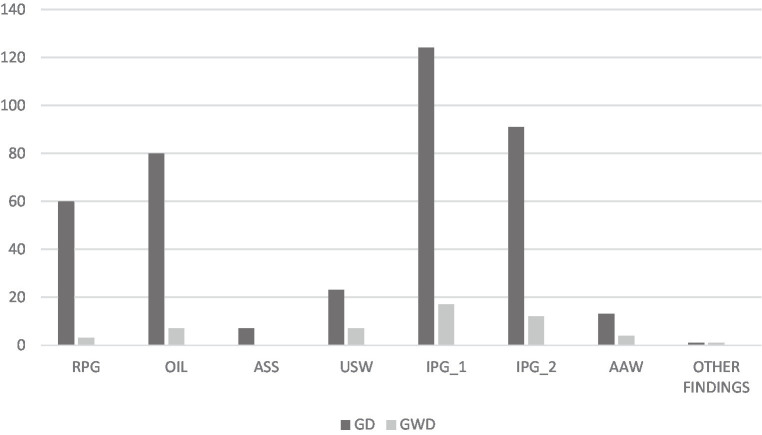
Number of different forms of error and semiological classification of spelling errors observed in the words written by GD and GWD. Regular phoneme/grapheme correspondence (RPG); Omission or insertion of letters (OIL); Alteration in the syllabic structure (ASS); Unconventional segmentation of word (USW); Irregular phoneme/grapheme correspondence type 1/Orthographic rule dependent of the context (IPG_1); Irregular phoneme/grapheme correspondence type 2/Orthographic rule independent of the context (IPG_2); Alteration in accentuation of words (AAW).

The target word *conseguiu/got*, for example, was written incorrectly 28 times and in 15 different forms by GD students. The students by GWD misspelled the target word *conseguiu/got* 11 times and in 4 different forms. Figure 2 presents the number of different forms of errors and the semiological classification of spelling errors observed in the words written by GD and GWD.

According to the semiological classification, GD students made 399 different forms of errors, 228 of which were arbitrary spelling (AS) and 171 were natural spelling (NS). The AS class with the most variety forms in GD was irregular phoneme/grapheme correspondence type 1 with 124 different forms of errors, followed by irregular phoneme/grapheme correspondence type 2 with 91 different forms of errors. Among the 171 NS different forms of errors in the GD, 80 different forms of errors were of the omission or insertion of letters type and 60 different forms of errors of the regular phoneme/grapheme correspondence type. The NS errors less observed in the GD were of the alteration in the syllabic structure type with 7 different forms of errors. The other designation includes letters with word stroke/illegibility problems; writing another word or writing an invented word—*pseudowords*; and writing numbers or drawings.

GWD students made 51 different forms of errors, 33 of which were AS and 18 were NS. The AS class with the most variety of forms in GWD was irregular phoneme/grapheme correspondence type 1 with 17 different forms of errors, followed by irregular phoneme/grapheme correspondence type 2 with 12 different forms of errors. Among the NS errors, the omission or insertion of letters and unconventional segmentation of words correspond to 7 different forms of errors each. In GWD, the alteration in the syllabic structure type NS error was not observed. There was one occurrence of others.

### 4.1 Analysis of word errors by GD and GWD

In this section, the types of errors of the target words will be analyzed and discussed in descending order as to the number of students in the GD who got them wrong. The numbering next to each word corresponds to its order in the ‘DSW’ list and will appear the first time it is cited in this section.

The target word ***de repente**/suddenly* (33), which is an adverbial locution composed of two words, was written incorrectly by 29 students from the GD and by nine from the GWD. The 38 students wrote it with the natural spelling error of the unconventional segmentation of word type, <*derepente* > and < *derrepente*>, joining the preposition <de> with the noun <repente>, indicating the influence of orality, contradicting the semiographic principle that words are not phonological units, but units defined by meaning and grammar (Nunes and Bryant, 2014). However, this separation does not seem to be obvious for students with dyslexia. Another natural spelling error made only by the GD was of the RPG type, in <terrepende>, in the exchange of the graphemes <d > for <t > and < t > for <d>, <direpente>, in the exchange of < e > for <i>, and < terepeite>, in the exchange of <d > for <t > and < n > for <i>. The exchanges between graphemes that represent homorganic phonemes (p/b, t/d, f/v, c/g, s/z, x/j), in turn, appear in the written productions of few students, even in the initial phase of literacy (Zorzi, 2003).

The target words ***a gente**/we* (25) and ***conseguiu**/got* (7) were tied for second place in terms of the number of students in the GD who got them wrong. ***Conseguiu**/got* had the highest number of different writings. In GD, 28 students spelled it in 15 ways, and 11 students from the GWD wrote it in four different ways, three in common with the GD. Perhaps the high rate of spelling errors is due to the fact that there is no regular syllable in this word. The first syllable presents rule situations both in the consonant and the nasal vowel, the second syllable is irregular, due to the possibilities of <se> and < ce> to spell /se/, and, in the third syllable, there is a contextual rule for spelling /gi/ and a morphological rule for the semivowel /w/. As far as ‘regularity’ is concerned, Portuguese seems to present fewer grapheme–phoneme irregularities than English, for example (Pinheiro, 1995).

Regular phoneme/grapheme correspondence type natural spelling errors appear in <*qu**a**nce**q**uio*>, <*consi**q**uil*>, <*conse**q**uil*>, and < *conce**q**uio*>, with exchanges of the graphemes <o > for <a > and < gu> for <qu>, and in <*cocegi**r***>, replacing <u > with <r>. The omission or insertion of letters error was seen in <*cosegiu*>, <*cocegiu*>, and < *cocegir*>, omitting <n > to nasalize the vowel /oN/ at the end of a syllable. Error of type IPG_1 occurred in: <***qu**ancequio*>, with exchange of <c > for <qu>; <*co**m**seguiu* > and < *co**m**se**g**iu*>, with exchange of <n > for <m>; <*conse**g**il*>, <*conse**g**io*>, <*cose**g**iu*>, <*coce**g**iu*>, <*coce**g**ir*>, and < *conse**g**iu*>, with the exchange of <gu> for <g>, in the writing of the verb in the simple past, in <*quancequi**o***>, <*consegi**l***>, <*consegui**o***>, <*concegui**o***>, <*consegi**o***>, <*consiqui**l***>, <*consegui**l***>, <*consequi**l***>, and < *concequi**o***>, with exchanges of <u > for <o > or < l>. The irregular phoneme/grapheme correspondence type 1 error was seen when the syllable <se> was changed to <ce>, in <*cons**i**quil*>, with the change of <e > for <i>, since the oral vowels /i/ and /u/, when pretonic, are irregular and can be spelled by <i – e > or < u – o > .

There were 28 students from the GD and 10 students from the GWD who wrote ***a gente**/we* incorrectly. The target word ***a gente**/we* is a pronominal locution that corresponds to two linguistic signs, being a definite article and a noun, but in orality, it can be confused with the noun <agente>. For natural spelling, the unconventional segmentation of word type was seen in all the errors of the 37 students with the junction of the target word, with the exception of a single form, in which the USW error was seen, with the separation in writing <*aj**e t**e*>, and the omission or insertion of letters error, in the omission of <n>. The regular phoneme/grapheme correspondence error was seen in <*aje**i**te*>, replacing <n > with <i>, and in <*agen**d**e*>, replacing <t > with <d>. Two types of AS errors occurred: IPG_1 appeared in <*agent**i***>, in the replacement of <e > by <i>, and IPG_2, in the errors <*a**je**nte*>, <*a**je**ite*>, and < *a**je** te*>, because the union of the phonemes /Ʒ/ with /e/ forming the syllable /Ʒe/, being an irregular situation, can be written both by <ge> and < je>. So, before <e > and < i > either <j > or < g > can arbitrarily occur (*gente/people, jeito/way*) and, as a result, the learner will have to memorize the words spelled with <g > and the ones spelled with <j > in this context. However, the existence of failures in the orthographic lexical storage in dyslexia is frequent and persistent throughout schooling (Goulandris, 2003; Angelelli et al., 2014).

The target words ***por isso**/therefore* (23) and ***ninguém**/nobody* (42) appear in third place, regarding the number of students from the GD who made mistakes in the dictation. All 33 students wrote ***por isso**/therefore* making the natural spelling error of the unconventional segmentation of word type, with a predominance of the merge, including the form <*porisso*>. The students without dyslexia spelled the phoneme /s/ of the word <isso> using the grapheme <ss>, probably applying their orthographic knowledge. From natural spelling, other errors appeared in GD, <*p**u**riso* > and < *por**ci**o*>, respectively, regular phoneme/grapheme correspondence type, in the exchange of the grapheme <o > for the grapheme <u>, and alteration in the syllabic structure type, in the inversion of the positions of the graphemes <i > and < c>. The arbitrary spelling error of type IPG_2 appears in the forms <*pori**s**o/puri**s**o* > and < *pori**ç**o*>, in the exchange of the grapheme <ss> for the graphemes <s > and < ç>, and also in the incorrect form <*por**c**io* > .

For the target word ***ninguém**/nobody*, 10 errors occurred, nine in the GD, and one in the GWD. A higher number of errors were observed in arbitrary spelling than in natural spelling, and the AAW type with the absence of acute accent was predominant for both groups. The irregular phoneme/grapheme correspondence type 2 error was observed in the inaccuracies <*ninge**n***>, <*nige**i***>, and < *minge**n*** > and the IPG_1 type error in the errors <*nin**g**en*>, <*ni**g**ei*>, <*min**g**en*>, and < *nin**g**em*>. For natural spelling errors, the omission or insertion of letters type was verified, with a predominance of omission of nasalization in /iN/ and /eN/, found in <*n**i**guem*>, <*ningu**e***>, <*n**i**gei*>, and < *ninqu**e***>; in addition to the RPG type for the exchange of graphemes <m > for <n > and < g > for <q > in the second syllable, in <***m**imgu**e**m* > and < *nin**q**uem*>. In the case of English, for example, there are no accents, and the tonic syllable is not marked. The readers need to use their lexical knowledge to know the strongest syllable in the word (Nunes and Bryant, 2014). In other languages, such as Portuguese, Spanish, and Greek, the strongest syllable is marked, considering some rules in its marking. It is important to emphasize that the marking of the tonic syllable is a concept that is not included in the idea of correspondences between letters and sounds because in the case of ‘pais’ *(fathers)* and ‘país’ *(country)*, the words are different and this difference is marked by the accent (Nunes and Bryant, 2014).

***Assalto**/assault* (27) presented 12 different incorrect spellings by 26 students from GD. Two students from the GWD wrote it in two ways in common with the GD, emphasizing that the errors occurred in the phonographemic conversions of the consonant phoneme /s/ and the semivowel /w/, given the high complexity of the syllabic structure of the second syllable of this target word (CCVC, where C denotes consonant and V denotes vowel) and its irregular condition. The two situations seen in the two groups included arbitrary spelling errors of the IPG_2 type in the inaccuracies <*a**s**alto* > and < *a**s**a**u**to* > .

The irregular phoneme/grapheme correspondence type 2 error, in which only the GD students wrote incorrectly, occurred in the forms <*a**ç**alto*>, <*a**uç**ato*>, <*a**us**ato*>, <*al**ç**ato*>, <*assa**u**to*>, and < *aca**u**to*>. This result shows the phoneme /s/ being the most complex for notation in Portuguese (Scliar-Cabral, 2003; Batista et al., 2014), as it can be represented in 10 ways: <s>, <c>, <x>, <z>, <ç>, <ss>, <sc>, <sç>, <xc>, and < xs>, which makes it difficult to choose which grapheme to use in a word that contains this phoneme. The graphemes <l > or < u > are also frequently observed to represent the semivowel /w/.

With regard to natural spelling, one highlights the type of alteration in the syllabic structure error in <*a**l**çato*>, <*a**u**çato*>, and < *a**u**sato>*, with the change in the order of the semivowel /w/ represented by the graphemes <l > and < u>, from the second to the first syllable, and < *assato**u***>, from the second to the last syllable. There was an error of type OIL, in the addition of grapheme <l > in <*a**l**caldo*>. The regular phoneme/grapheme correspondence type error appeared in the exchanges of the graphemes <ç > for <c > and < t > for <d>, in <*a**c**auto* > and < *al**c**al**d**o*>. The NS errors occurred only in the GD.

The target word ***alguém**/somebody* (48) was written incorrectly by 26 students from each group, GD and GWD. Despite the high complexity of the syllabic structure of the last syllable, when compared to the target word ***assalto**/assault*, fewer incorrect forms were observed, five for the GD and one for the GWD. The target word ***alguém**/somebody* contains the nasal vowel /eN/ in the final syllable of a word that is not a verb, produced orally as [eNj], generating the condition for arbitrariness in its notation. Most of the incorrectly spelled forms belong to the AAW type error of arbitrary spelling with the absence of accentuation, including the incorrect form <*algu**e**m* > from GWD. Other incorrect forms of arbitrary spelling, all from GD, revealed that the complexity of the last syllable can justify the following errors: type IPG_1 in <*al**g***e*m*>, <*au**g**en*>, and < *am**g**en*>, in which the digraph was not preserved; type IPG_2 in <*a**u**ge**n***>, <*amge**n***>, and < *alqe**n***>. The errors in <*a**m**gen*>, with the exchange of the grapheme <l > for the grapheme <m>, <*al**q**en*>, and < *al**q**uém*>, with the exchange of the grapheme <g > for the <que>, characterize the type RPG of the NS.

In the fifth position, the target word ***exame**/exam* (53) was spelled incorrectly by 24 students from the GD, with six types of writing, and by only four students from the GWD, with two incorrect forms. Natural spelling errors of the omission or insertion of letters type occurred only in the GD, with the addition of the grapheme <i > in the following forms: <*eza**i**me* > and < *exa**i**me*>. There was a predominance of spelling errors of AS type IPG_1 for both groups. The inaccuracies <*e**z**am**i***>, <*e**s**am**i***>, <*i**s**am**i***>, <*e**s**ame*>, and < *e**z**aime* > occurred in the GD and < *exam**i*** > in the GWD. Finally, the spelling error < ***i**sami*>, presented in the GD, is of the irregular phoneme/grapheme correspondence type 2 error.

Twenty-three students misspelled the target words ***embaixo**/under* (35) and ***fez**/did* (40), and 22 got wrong ***em cima**/above* (34) and ***faz**/does* (38), all from GD. In the GWD, six students got the wrong ***em cima**/above* and ***embaixo**/under,* and one student got the wrong ***fez**/did* and ***faz**/does*. Although they are short words, the error may lie in the irregularity of the archiphoneme /S/ in the final position of the word in stressed monosyllables, in the decision between the graphemes <s > and < z>. This situation was observed in the incorrectness of the IPG_2 type of the AS in <*fai**s***>, <*fa**s***>, and < *fei**s***>. Only one type of NS error was presented by the addition of graphemes in the GD and GWD groups in these target words. The forms found were < *fa**i**s*>, <*fe**i**s*>, <*fe**i**z**e***>, and < *fe**i**z* > .

The target word ***em cima**/above* was written incorrectly by GD and GWD, with six and two forms, respectively, and all with an unconventional segmentation of word type error with word junction: <*e**ns**ima*>, <*e**mc**ima*>, <*i**nc**ima*>, <*e**nss**ima*>, <*e**nc**ina*>, <*e**mic**ima*>, <***em**cima>,* and < *e**ms**ima*>. This error gave rise to another (type IPG_1) when the students ignored the fact that ***em cima**/above* is an expression formed by two words that constitute a prepositional phrase, writing <***in**cima*>, <***en**sima*>, <***en**ssima*>, and < *e**nc**ina*>. Other errors made only by the GD were the RPG type, in <*enci**n**a*>, with the replacement of <m > by <n>, and the omission or insertion of letters type error, with the addition of <i > in <*em**i**cima*>. The same error of type IPG_2 composed the three incorrect forms: for the GD, <*en**s**ima* > and < *en**ss**ima*>, and for the GWD, it was <*em**s**ima*>, due to the possibilities of <si> and < ci> to note /si/.

The target word ***embaixo**/under* appears with seven errors for the GD and one for the GWD. Unconventional segmentation of word type natural spelling errors occurred in the GWD in <*em baixo* > and in the GD in <*em baicho*>. In the GD, the error type regular phoneme/grapheme correspondence was observed in <*embai**s**o*>, <*embai**j**o*>, and < *enba**ç**o* > when replacing the grapheme <x > with the graphemes <s>, <j>, and < ç>. The OIL type error was considered in the errors <*enbaço* > and < *ebaixo*>, with the omissions of the grapheme <i > when forming the diphthong and the grapheme <m > in the nasalization of the vowel /eN/. The arbitrary spelling type IPG_1 appears in <*embai**ch**o*>, <*e**n**baço*>, <*em bai**ch**o*>, and < *e**n**bai**chu*** > .

The target word ***também**/also* (4) was written incorrectly by five students from the GWD, but all of them made the same arbitrary spelling error, type alteration in accentuation of words, without the acute accent in the grapheme <eN>. In all errors, both GWD and GD made the alteration in accentuation of words type error. It is therefore recommended that schools invest in teaching graphic accents. ***Também**/also* contains the nasal vowel /eN/ in the final syllable of a word that is not a verb, produced orally as [eNj], leading to a situation of arbitrariness in its orthographic notation. IPG_2 type AS error was observed in <tabei> and < tanbei> in GD. GD schoolchildren, when replacing <m > with <n > to nasalize the vowel /aN/ at the end of an internal syllable, in the writings of <*ta**n**be*>, <*ta**n**pem*>, <*ta**n**bei*>, and < *ta**n**bem*>, made the arbitrary spelling error of the irregular phoneme/grapheme correspondence type 1. RPG type natural spelling errors were seen in <*tan**p**em* > and < ***d**am**p**em* > by replacing the graphemes <b > with <p > and < t > with <d>; and the spelling error type omission or insertion of letters when omitting the grapheme <m > in the spellings <*tanbe*>, <*tabem*>, and < *tabei*>. Bodard et al. (2023) reviewed studies that classified spelling errors produced by dyslexics from texts in English (Pedler, 2007), Spanish (Rello et al., 2012, 2014), German (Rauschenberger et al., 2016), and French (Antoine et al., 2019). Based on that analysis, they proposed a new study of spelling mistakes made by French dyslexics. In the results, errors were also observed due to changes in the graphemes <b > and < p > and < t > and < d>, classified as ‘Confusion between graphemes phonetically close.’

The target words ***exemplo**/example* (11), ***enxergar**/see* (36), ***guerra**/war* (10), and ***caiu**/fell* (31) were tied for 8th place in terms of the number of students in the GD who got them wrong. The target word ***exemplo**/example* was written with the second highest number of inaccuracies in the entire list. Altogether, 14 forms were observed, with 21 students from the GD writing ***exemplo**/example* with 13 errors and two GWD students with an incorrect form. There was a greater number of errors in AS compared to those in NS, which can be explained by the syllabic complexity of the word. The second syllable <xem> must be spelled with the grapheme <x > .

For arbitrary spelling, the biggest problem was in writing the second syllable to spell the phoneme /z/ when <x > was replaced by <s > and < z>, configuring the error of type IPG_1, in the GD errors: <*e**s**enplo*>, <*e**s**enplu*>, <*e**s**eplo*>, <*e**s**emplo*>, <*e**z**enplu*>, <*e**z**enplo*>, <*e**z**enpo*>, <*e**z**eblo*>, and < *e**z**emplo*>. Still, in the second syllable, the students from the GD misspelled the nasalized vowel, characterizing the IPG_1 error: <*eze**n**plu*>, <*ese**n**plo*>, <*ese**n**plu*>, <*eze**n**po*>, <*ege**n**po*>, and < *eze**n**plo*>, while two students from the GWD presented only the error < *exe**n**plo*>. This same type of IPG_1 error was observed in arbitrary spelling in <*ezenpl**u*** > and < *esenpl**u***>. As for the natural spelling errors, two types appeared, the regular phoneme/grapheme correspondence type, in <*eze**b**lo* > and < *e**g**enpo*>, and the OIL type, with grapheme omissions in <*exmplo*>, <*eseplo*>, <*ezenpo*>, <*ezeblo*>, and < *egenpo*>, followed by the same type omission or insertion of letters with the addition of graphemes in <*ex**s**emplo* > and < *e**n**xemplo* > .

***Enxergar***/*see* was the target word spelled incorrectly by 21 students from GD and six students from GWD. In the word ***enxergar***/*see,* there was an error, for both groups, in <*e**m**xergar* > (IPG_1 type error). The same type of error appeared for both groups in <*en**ch**ergar* > and for the GD in <*en**ch**ega*>. Regular phoneme/grapheme correspondence type errors were seen in <*en**j**ergar*>, of the GWD, and < *en**j**e**i**ga*>, <*en**s**erga*>, and < *en**ç**ega*>, of the GD, pointing out to the fact that the phoneme /ʃ/ can be a challenge in the spelling of dyslexics, since <j>, <s>, and < ç > were chosen for the notation of <x>, as well as in the errors <embai**s**o>, <embai**j**o>, <borra**j**a>, <bru**j**a>, and < bru**ç**a>, reinforcing that errors related to natural spelling have a high incidence in the dysorthographic profile (Batista et al., 2014). In ***enxergar**/see*, GD students made errors of the omission or insertion of letters type in the forms <*enchega*>, <*enjeiga*>, <*enserga*>, and < *ençega*>, with a predominance of the omission of the grapheme <r > to signal the verb in the infinitive. The consonant digraph <ch> is considered by Nunes and Bryant (2014) as an ‘extra’ because the sound it represents can be played by the letter <x>, which is not a consistent function letter and certainly causes problems for the student. In English, for example, the letter <f > could be used in all words that represent the sound /f/, but it is not: ‘elephant,’ ‘telephone,’ and ‘laugh.’ According to Faraco (1992), the letter <x > is always used after diphthongs, in words of indigenous or African origin, and after the initial syllable <en>, with few exceptions. However, despite these regularities, the representation of the sound performed by the <ch> digraph is an example of unpredictable spelling (Nunes and Bryant, 2014), because many cases are not resolved by the mentioned rules, for example: ‘chave’ (*key*), ‘xadrez’ (*chess*), and ‘macho’ (*male*).

Regarding the target word ***guerra**/war*, 21 students from the GD and one student from the GWD got it wrong. The natural spelling error type RPG appears once in the GD when replacing the digraph <gu> with <qu> in <***qu**erra* > and < ***qu**era*>. A spelling error of the type IPG_1 of arbitrary spelling was observed in the GD, in the writings <*que**r**a*>, <*gue**r**a*>, and < *ge**r**a*>, with the omission of a grapheme <r > in the composition of the digraph. The students from the GD also presented the irregular phoneme/grapheme correspondence type 1 error of arbitrary spelling in <***g**erra* > .

In the case of the word ***caiu**/fell,* as it is a verb in the simple past, the semivowel [w] at the end of a word acts as a morpheme and must be spelled with <u>. The 21 students from the GD presented arbitrary spelling errors of the IPG_1 and alteration in accentuation of word types in their writing. For the first type, the following inaccuracies were found in <*cai**o*** > and < *caí**o***>, with the grapheme <o > being used. In the second type, one sees <*ca**í**o*>, with the alteration in accentuation of words. None of the GWD students wrote the word ***caiu**/fell* incorrectly. Stress marking involves the differentiation between vowels and semivowels, mainly at the end of words, when we use different letters to mark /u/ as a vowel and /w/ as a semivowel (Nunes and Bryant, 2014).

The target words ***começou**/started* (32) and ***almoço**/lunch* (26) appear in ninth place, with 20 being the number of students from the GD who made mistakes in the dictation. As for the GWD, one student misspelled the word target ***começou**/started* and two schoolchildren made a mistake in ***almoço**/lunch*.

When writing the target word ***almoço**/lunch*, the students of both groups made all incorrect spelling errors of arbitrary spelling of the type IPG_2 in the irregular syllables <al> and < ço>, replacing them with <au> and < so>, sometimes in one, sometimes in another, or in both. The incorrect form <*almo**s**o* > was verified in GD and GWD. The spelling error IPG_2 was observed in the GD in <*almou**s**o*>, <*a**u**mo**s**o*>, <*a**u**moço*>, and < *a**u** moço*>. A curious fact is that none of the students used <ss> in the last syllable, which would also characterize the error; however, it would not change the graphophonemic relationship in the reading of the target word. Two types of natural spelling errors appeared in the GD writing: the omission or insertion of letters type error, with the addition of the grapheme <u > in <*almo**u**so*>, and the USW type, in the variant <*au moço*>. There was also the form <*almoção*>, classified as others.

The incorrect form shared by GD and GWD for the target word ***começou**/started* was <*come**s**ou*>, characterizing an arbitrary spelling error of type IPG_2 in the irregular syllable <çou>. Five forms were used by the GD. The arbitrary spelling IPG_2 type error was seen in <*come**ss**ou*>, <*come**s**ol*>, and < *comei**s**ou* > when errors appear in the exchange of <ç > for the graphemes <s > and < ss>. Still from AS, however, of the IPG_1 type, there is the form <*comeso**l***>. The occurrence of this error has already been discussed in the target words ***caiu**/fell* and ***conseguiu**/got*. As for natural spelling, the GD showed regular phoneme/grapheme correspondence and omission or insertion of letters types of errors. For the first type, <*come**c**o* > and < *come**c**ou*>, without the cedilla diacritical in <c>. In the second type, OIL, there was insertion in <*come**i**sou* > and omission in <*comeco* > .

The target word ***vez**/time* (5) was spelled correctly by all GWD students, and 19 GD students spelled the target word incorrectly: <***f**ez*>, <*ve**i**z*>, <*ve**is***>, and < *ve**s***>. For the first occurrence, the natural spelling RPG type error was observed in the replacement of <v > by <*f*>. For the second and third occurrences, the omission or insertion of letters type error was seen by adding the grapheme <i>. The error type IPG_2 was identified in the substitution of the grapheme <z > for the <s > in the coda or post-vocalic position when writing <*vei**s*** > and < *ve**s*** > .

As in the previous target word, ***vez**/time*, the target word ***relógio**/clock* (56) appears tied for 10th position in the number of students who made a mistake, with 19 students from the GD and six from the GWD. In all of them, the arbitrary spelling error of the alteration in accentuation of words type appeared, with the absence of the acute accent, which was the only inaccuracy for the students of the GWD, in <*rel**o**gio*>. GD students also made other arbitrary spelling IPG_2 type errors in <*relo**j**o*>; of NS of type regular phoneme/grapheme correspondence in <*relo**z**o*>, and of type omission or insertion of letters, with omission of the grapheme <i > in <*relojo* > and < *relozo* > .

In relation to the target word ***jeito**/way* (22), four distinct forms of notation were verified for 18 students from the GD. A GWD student made the same arbitrary spelling error, type IPG_2, expressed in <***g**eito*>, confirming that the spelling of irregular situations can be a complication for the student. The GD showed other incorrect spellings of arbitrary spelling of the types IPG_2, when writing the phoneme /Ʒ/, and IPG_1, violating a contextual rule in <***g**eit**u***>, respectively. Types RPG occurred in <*je**n**to*>, with an exchange of <i > for <n>, and OIL, in <*jeto*>, with omission of <i > from NS in inaccuracies by GD.

The target words ***disse**/said* (3) and ***árvore**/tree* (50) were written incorrectly by 17 students from the GD and by two students from the GWD, occupying a 12th place in the ‘DSW’ list. According to the semiographic principle (Marec-Breton and Gombert, 2004), an arbitrary spelling error of irregular phoneme/grapheme correspondence type 1 occurs when writing from the morpheme (minimum unit capable of expressing meaning), and not from the phoneme, fails. Thus, it was observed, regarding the target word ***disse**/said*, in the incorrect notation forms <*di**ce***>, <*di**ci***>, and < *di**se***>, and even in <*di**çe***>, a transgression of the Portuguese orthography for the students of the GD.

Few inaccuracies were observed for the target word ***árvore**/tree*. The GWD presented the alteration in accentuation of words type of error in <***a**rvore*>, with the absence of graphic accent on the stressed syllable. GD also made the same mistake as GWD in <***a**vori*>, with the absence of the acute accent, and in <***a**rvor**é***>, with the inadequate presence of the acute accent, showing that, even with the rule that all proparoxytone words must be accented, it is not used by all students. The replacement of <e > by <i > characterizes the same class of error as arbitrary spelling, but of irregular phoneme/grapheme correspondence type 2, in the form <*avor**i***>, and may occur due to failure to activate the orthographic lexical memory (Batista and Capellini, 2011), which brings the mental representation of the orthographic patterns expected for each word with irregularity, since generally the posttonic oral unstressed vowels /i/ and /u/ convert to <e – i > and < o – u>, being the most frequent spellings <e > and < o > (Scliar-Cabral, 2003). There were also the forms <*ávore* > and < *avori* > for the GD, with the omission of the grapheme <r>, an NS error of the omission or insertion of letters type.

The target words ***saudade**/longing* (57), ***pegue**/take* (43), and ***borracha**/rubber* (28) were spelled incorrectly by 16 students from the GD. For the first two, there were four forms, and for the last one, seven. As for the number of students from the GWD with poor performance for the writing of the target word ***saudade**/longing*, there were three, while for ***pegue***/*take* and ***borracha**/rubber,* it was just a student presenting a form for each target word.

The highest error occurrence for the target word ***saudade***/*longing* was the writing of the first syllable, as there is a rule situation (<sa > starting the word) and an irregularity situation (the semivowel notation /w/, which can be written by <l > or < u>). Errors of irregular phoneme/grapheme correspondence type 1 appeared in <*sa**l**dade* > for both groups and < *sa**l**tade* > for the GD; the error of irregular phoneme/grapheme correspondence type 1, in <***ç**audade*>, just for GD. In Portuguese, there are also rules of position: the ‘doubled’ letters and the <ç > are not used at the beginning of words. The rule is only of form because <rr>, <ss>, and < ç > would be read the same way if they appeared at the beginning of the word (Nunes and Bryant, 2014). GD also presented a natural spelling error of regular phoneme/grapheme correspondence type in <*sal**t**ade*>, with the exchange of <d > for <t>, and < ***c**audade*>, by replacing <s > with <c > .

All students who misspelled the target word ***pegue**/take* coincided in the second syllable, in the two situations of contextual rule <**gu**e > and < gu**e**>, making IPG_1 type arbitrary spelling errors. GD and GWD shared the same occurrence, <*pe**g**e*>. The errors <*pe**gi*** > and < *pegu**i*** > occurred only in the GD. This group also made a type of natural spelling error, regular phoneme/grapheme correspondence, in <*pe**q**ue*>, replacing the grapheme <g > with <q > in the formation of the digraph.

More than half of GD students wrote the word ***borracha**/rubber* (28) incorrectly. For natural spelling, there were the types of errors in regular phoneme/grapheme correspondence, seen in <*borra**j**a*>, <*bora**j**a*>, <*bora**s**a*>, and < *borai**j**a*>, with exchanges of the grapheme <ch> for the graphemes <j > and < s>, transforming the target word into a *pseudoword*. In <*bora**i**ja*>, the type omission or insertion of letters also appears, with the addition of the grapheme <i>. Examples that indicate arbitrary spelling errors are irregular phoneme/grapheme correspondence type 1 and irregular phoneme/grapheme correspondence type 2, which appear in <*bo**r**aja*>, <*bo**r**axa*>, and < *bo**r**asa*>, with the contextual exchange of the digraph <rr> for the grapheme <r>; <*bora**x**a*>, and < *borra**x**a*>, with the error in the irregular syllable <cha>, since the phoneme /ʃ/ accepts two spelling possibilities, <x > and < ch>. The form <*bo**r**acha* > has error type IPG_1, presented by GD and GWD. Rego and Buarque (1996) observed that the trajectory of students in learning the use of <r > and < rr> cannot be described as a simple matter of using rules learned simultaneously. Many students in the early years use only the <r>, perhaps because they are basing their spellings on the idea that a single letter is written for each sound.

Half of the GD made errors in the target words ***assim/**so* (13) and ***amanhã**/ tomorrow* (49), and in the GWD, only one and three students, respectively, spelled them incorrectly. For the word ***assim/**so,* the omission or insertion of letters error was seen in <*aci* > and < *assi* > when there was the omission of <m > for nasalization of the vowel /iN/. Even in more advanced students, from GD5 and GD6, errors of the IPG_2 type were observed when spelling /s/, in <*a**c**im*>, <*a**c**i*>, and < *a**s**im*>, demonstrating that problems in the consolidation and use of orthographic lexical memory (Apel et al., 2019) are common in schoolchildren with dyslexia. Only one student from GWD made an error of irregular phoneme/grapheme correspondence type 1 in <*assi**n***>, nasalizing the vowel with <n > .

When spelling the target word ***amanhã***/*tomorrow* incorrectly, 15 students from the GD and three students from the GWD had the same IPG_1 error in <*amanh**a***>, with the absence of the tilde to indicate nasalization, also observed in the GD, in the forms <*amanha**m*** > and < *amaha**n***>. Alteration in the syllabic structure type error occurred with alteration of the last syllable of the target word ***amanhã/**tomorrow*, being written as <*ama**han*** > in GD.

The target word ***correr/**run* (52) had two incorrect modes: <*corre* > and < *co**r**er*>. The first is an omission or insertion of letters type error, with omission of <r > in both groups. There is also an arbitrary spelling error of irregular phoneme/grapheme correspondence type 1, with the use of <r > in the constitution of the digraph <rr> in the GD. There were 14 students from the GD and three from the GWD who got it wrong, positioning ***corer/**run* in 15th place in the occurrence of misspelled words.

The target words ***brincar***/*play* (14) and ***homem**/man* (21) appear in 16th place, with 13 being the total number of students in the GD who got them wrong. A GWD student got ***homem**/man* wrong, making the irregular phoneme/grapheme correspondence type 2 error when writing the nasalized vowel /eN/ at the end of a word, using <n > instead of <m>. GD students made the same IPG_2 error in: <*home**n***>, <*homi**n***>, <*home**i***>, <*ome**n***>, and < *ome**i***>. The IPG_2 error also appears in the omissions of <h > in <*omem*>, <*omen*>, and < *omei*>. Another omission of a segment arose in <*home*>, without using the grapheme <m>; however, in this situation, configuring an NS error of type omission or insertion of letters of natural spelling, the error of type regular phoneme/grapheme correspondence appears in <*hom**i**n* > .

Only GD students had errors in the target word ***brincar**/play*, and only in natural spelling, predominantly of the omission or insertion of letters type, with omissions of graphemes, in <*brica*>, <*bincar*>, <*binca*>, <*bicar*>, and < *brinca*>. It is important to emphasize the structures of the two syllables that form the target word. In the first, there is CCVC, and in the second, CVC. Syllable structures that deviate from the canonical CV pattern generally offer more challenges for students with dyslexia, as they demand more cognitive–linguistic actions at the time of writing. There is a natural spelling error of type RPG in <***p**rincar*>, in which there is an erasure of the sonority that the grapheme <b > presents.

Less than half of the students in the GD wrote the target words ***gente**/people* (9), ***viajar**/travel* (24), and ***certo**/right* (51) incorrectly. The target word ***gente**/people* was written orthographically by all GWD students. In the GD, 12 students wrote it incorrectly, with five forms. Natural spelling errors arose in <*jen**d**e*>, marking the regular phoneme/grapheme correspondence type, replacing <t > with <d>; in <*j**e**te* > and < *j**e**ti*>, characterizing the omission or insertion of letters type, with the omission of nasalization of vowels in writing. In all misspelled, there were AS errors. The IPG_1 type appeared in <gent**i** > and < *jet**i***>, violating the contextual orthographic rule because when the stress of the word falls on the penultimate syllable, the vowel phoneme /i/ must be written with the grapheme <e>. The IPG_2 type error appeared when choosing the grapheme <j > instead of <g>: <***j**ete*>, <***j**eti*>, <***j**ente*>, and < ***j**ende* > .

One of the words that GWD misspelled just once was ***viajar**/travel*. Twelve students from the GD wrote this word in the same way as the GWD, presenting an OIL error, omitting <r > in <*viaja*>. GD students replaced the grapheme <j > with <g>, perhaps taking into account the acrophonic principle, focusing on the AS IPG_1 type error in the form <*via**g**ar* > .

With arbitrary spelling errors predominating, the target word ***certo**/right* was written incorrectly by GD students in two ways. The irregular phoneme/grapheme correspondence type 2 error appeared in <***s**erto* > and < **s**ertu > due to the possibility of using the graphemes <se> or < ce> to write the syllable /se/. The second form showed the error of type IPG_1 in <sert**u**>. GWD did not present an error in this target word.

All GWD students correctly spelled the target word ***encontrou**/found* (8), and 11 students from the GD wrote it incorrectly, in nine different ways. The superiority of spelling errors in arbitrary spelling was observed, possibly resulting from the absence of simple and regular syllables in the target word. Interestingly, there was no omission of nasalization in the syllables /eN/ and /koN/, but the transgression of the contextual rule with the substitution of the grapheme <n > for <m>, characterizing the error of the type IPG_1, in the forms <*e**m**controu*>, <*enco**m**trou*>, <*enco**m**trol*>, and < *e**m**co**m**trol*>. To know whether the nasalization of a vowel in the middle of the word should be done with <m > or < n>, students need to look at the next syllable. For example, in the word ‘bomba’ (*bomb*) the nasalization of the <o > is done with <m > because the following syllable starts with <b>, and in the word ‘conde’ (*earl*), the nasal marking is done with < n > because the following syllable starts with <d>, and not with <b > or < p >  (Rego and Buarque, 1996).

The GD students also showed changes in the spelling rule of morphosyntax, with the irregular phoneme/grapheme correspondence type 1 error, for the written representation of the semivowel /w/ of the verb in the simple past, using the grapheme <u>, with the following incorrect words: <*encontro**l***>, <*encomtro**l***>, <*emcomtro**l***>, and < *incontro**l***>. The last type of arbitrary spelling error found was the IPG_2, observed in the errors of <***i**ncontrou* > and < ***i**ncontrol*>, with the exchange of the grapheme <e > for the grapheme <i>, in which there is no rule for the spelling of the pre-stressed unstressed vowel, which can be converted into <i > or < e>. Regarding NS errors, the OIL type was presented with the omission of the semivowel representation /w/ in <*encontro* > and < *encontor*>, besides the type ASS in this last form shown with the alteration in the syllabic structure of the syllable <trou> by the writing of <tor>.

The target word ***quente**/hot* (46) appeared with five errors, with 11 students from GD writing it in four ways and one student from GWD writing it incorrectly. There was a prevalence of the IPG_1 type error of arbitrary spelling in the students of the GD: <***q**ent**i***>, <***q**ente*>, and < *quent**i***>. The errors occurred in the two syllables of the word, with the simplification of the digraph <qu> and with the change of the grapheme <e > for <i>. GD and GWD presented natural spelling errors of the omission or insertion of letters type, with the addition of the grapheme <i > in the form <*que**i**nte* > for the first group, and of type RPG, in the error < ***g**uente*>, replacing the grapheme <q > by the grapheme <g > in the formation of the digraph, for the second group of students.

From now on, the analysis of the last third of the ‘DSW’ list will be conducted with the occurrences of words written incorrectly by the GD students, meaning that 10 or less students misspelled the next target words. A decrease in the number of incorrect forms is observed for each target word. Of the remaining one-third, GWD students made errors only in the target words ***futebol**/soccer* (15), ***tenho**/have* (47), ***tempo**/time* (58), ***então***/*then* (20), and ***muito**/much* (1).

Ten GD students misspelled the target words ***cabeça**/head* (30) and ***professora**/teacher* (45), with three errors for the first and one error for the second. The IPG_2 type AS error was seen in <*cabe**s**a*>, <*cabe**ss**a*>, and < *profe**s**ora*>. There was an incorrect spelling of the syllables <ça> and < sso> in the error observed in <*cabe**c**a*>, in GD. This result shows, once again, the phoneme /s/ is the most complex for notation in Portuguese (Scliar-Cabral, 2003; Batista et al., 2014).

The target words ***feliz**/happy* (54) and ***futebol***/*soccer* occupied the 20th place in terms of the number of students in the GD who made mistakes. The only mistake made by the GD when writing ***feliz**/happy* was the arbitrary spelling error of type IPG_2, with the replacement of <z > by <s > in the coda or post-vocalic position in <*feli**s***>. Although ***futebol**/soccer* is a frequent word in the Portuguese language, seven incorrect forms were seen, in addition to the influence of the English spelling made by a GWD student, in <*futball*>, being classified as others. Nine students from the GD and two students from the GWD presented six forms of spelling errors. The biggest problem occurred in the choice of graphemes to write the vowel phonemes /u/ in the first syllable, /e/ in the second syllable, and the semivowel /w/ in the last syllable; the first two, as they are in pretonic position, can be spelled with <o–u > and < e–i>, and the semivowel allowing the use of <u > or < l>. The arbitrary spelling errors of the IPG_2 type seen in both groups appeared in <*f**o**tebol* > and in GD < *fut**i**bol*>, <*fut**i**bo**u***>, and < *futebo**u***>. One more type of error, OIL of NS, was made by the GD and the GWD in the occurrence <*fu**l**tebol*>, with the addition of <l>, and only by GD in <*futebol**l*** > .

The target words ***tenho**/have*, ***tempo**/time*, ***tesoura**/scissors*, ***fazer**/do* (39), and ***ajuda***/*help* (12) occupy the 21st position in terms of the number of students in GD who made mistakes. Of these words, a GWD student spelled ***tenho**/have* incorrectly, three GWD students misspelled ***tempo**/time*, and eight students from GD presented incorrect notation in all target words in that block. The target word ***tenho**/have* was written incorrectly by GWD in <*tenho**r***>, characterizing the error of type omission or insertion of letters, with addition of <r>. GD presented the same type of error, with addition of <i > in <*te**i**nho*>, probably motivated by the influence of the oral articulation of the word and omission of the digraph <nh> in <teo>. Some GD students also spelled it as <*temum*>, orthographic occurrence classified as others, and also with the error < *te**iu***>, which presents two errors, one of natural spelling of regular phoneme/grapheme correspondence error, with the replacement of the digraph by <i>, and another error of arbitrary spelling of type IPG_1, using <u > instead of <o>, violating the contextual rule of the paroxytones ending with the phoneme /u/ which is written with <o > .

Both groups presented the incorrect form <*te**n**po* > for the target word ***tempo**/time*, with the arbitrary spelling error of type IPG_1 because the grapheme <m > should be used to write the nasal vowels before the graphemes <p > and < b>. Of the same type of error, GD presented the form <*te**n**p**u***>. The omission or insertion of letters error appeared in <*tepo*>, with the omission of the grapheme <m > for the nasalization of the vowel. It is observed, once again, that students can build syllabic structures that are not part of the Portuguese language, such as <npo> and < npu>. For the word ***tesoura**/scissors* (59), GD students showed two dysorthograph forms; however, in one of them, there were three errors: <***d**ezora* > presented two natural spelling errors, of type regular phoneme/grapheme correspondence, with the replacement of <t > for <d>, and omission or insertion of letters, with omission of <u>, in addition to the error of arbitrary spelling of type IPG_2, in the replacement of <s > by <z>. This type of error was also manifested in <*te**z**oura* > .

In the target word ***ajuda**/help*, GD students replaced the grapheme <j > with <g>, making the IPG_1 AS error in <*a**g**uda*>, as they did in ***viajar**/travel*. They presented spelling errors of natural spelling of the type regular phoneme/grapheme correspondence in the writing of <*a**x**uda*>, with the exchange of the grapheme <j > for the grapheme <x>, and of type OIL, in <*ajuda**r***>, with the addition of the grapheme <r > at the end of the word.

Three errors were presented by GD in ***fazer**/do*, two of natural spelling and one of arbitrary spelling. Omission of <r > at the end of the word occurred—OIL type error, as well as in all target words of the ‘DSW’ list that brought the nominal form of the verbs: ***corer**/run*, ***enxergar/**see*, ***brincar/**play,* and ***viajar/**travel*. The second NS error was of the regular phoneme/grapheme correspondence type in <***v**azer*>. The IPG_2 type error was seen in <*fa**s**er* > because it is a spelling irregularity for the notation of the phoneme /z/ between two vowels.

The target word ***muito/**much* was written in three incorrect ways by seven students in GD: <*mui**n**to*>, <*mu**n**ito*>, and < *muit**u***>; and by a GWD student sharing the first cited form. The addition of <n>, in any position of the word, is an omission or insertion of letters error, which may show that the writing of the vowel phoneme /i/, which sounds nasal in the oral pronunciation due to its proximity to the phoneme /m/, can lead the to described error. In GD, the arbitrary spelling error of type IPG_1 was also observed when replacing the grapheme <o > with <u > in <*muit**u*** > .

In the target words ***então/**then*, ***quando/**when* (2), ***cachorro/**dog* (6), and ***presente/**gift* (55), the number of students from the GD who got them wrong was the same, six. In the target word ***então/**then*, two GWD students made the same IPG_1 error, in <*e**m**tão*>, which requires the use of <n > to write nasal vowels before graphemes other than <p > and < b>. GD students also made arbitrary spelling errors, but different from GWD. The IPG_1 type was seen in <*ent**am*** > and < *int**au***>, indicating that the spelling of the morpheme [ãw] is an error. The IPG_2 type error was seen in <***i**ntau*>, with the exchange of <e > for <i>, suggesting that the spelling of the oral or nasal vowel phonemes /e/, /eN/, /i/, and /iN/ is challenging, especially if the syllable is unstressed. In the target word ***então/**then*, there is an error of type RPG, with the use of <i > instead of <n > in <*e**i**tão* > .

In the target word ***quando/**when*, the error occurred in the first syllable, formed by the CVVC structure. In longer syllabic compositions, natural spelling errors often occur, and in this word there were two types: regular phoneme/grapheme correspondence and omission or insertion of letters. In the case of <***g**uando* > and < ***g**ando*>, with the replacement of <q > by <g>, and also in <***can**do*>, the error may be due to a way in which the student says this word, changing /kuaN/ to /kaN/, characterizing the regular phoneme/grapheme correspondence error. The OIL type appears in the errors <*gando* > and < *cuado*>, with the omission of <u > and < n>, respectively. Mastery in the use of digraphs is preceded by a phase in which the student understands that the two spellings exist, the letters <c > and < g > and the digraphs <qu> and < gu>, but still do not know how to choose between the simple letter and the digraph depending on the context (Nunes and Bryant, 2014). The errors of arbitrary spelling that were made by the students of the GD appear in <*qua**m**do*>, with the type IPG_1, when <n > is replaced by <m>, to indicate nasality, and < ***cu**ado*>, with the type IPG_2, since the syllables <QU> and < CU> are irregular in the phonographemic conversion.

For the target words ***cachorro/**dog* and ***presente/**gift*, AS errors predominated, made only by students from the GD. The errors were of the IPG_1 type, in <*cacho**r**o* > and < *caxo**r**o*>, referring to the <rr> digraph, and of type IPG_2, in <*ca**x**oro*>, by the irregularity in the writing of the phoneme /ʃ/. The target word ***presente/**gift* was written in three ways, with errors in the medial and final syllables. The IPG_1 type error appeared in <*prezent**i*** > by replacing <e > with <i>, as well as in ***gente/**people*, ***a gente/**we*, ***pegue/**take*, ***quente/**hot,* and ***exame/**exam*, but not in ***saudade/**longing* and ***de repente/**suddenly.* The IPG_2 type error appeared in all modes, <*pre**z**enti*>, <*pre**z**ente*>, and < *pre**z**ete*>, in replacing <s > with <z>. A natural spelling omission or insertion of letters type error occurred in <*prezete*>, in which the <n > of the nasalized vowel was omitted.

Five students from the GD presented the type of spelling error regular phoneme/grapheme correspondence in the target word ***bruxa/**witch* (29), observed in <*bru**ç**a*>, <*bru**j**a*>, and < *brui**j**a*>, with the replacement of the grapheme <x > by the graphemes <ç > and < j>. Still, from natural spelling, the OIL type error was considered in the error < *bru**i**ja*>, with the addition of the grapheme <i>. The IPG_2 type was observed in <*bru**ch**a*>, in the change of the grapheme <x > for the grapheme <ch>, because the phoneme /ʃ/ accepts these two forms of writing.

In GD, four students incorrectly wrote the target words ***animal***/*animal* (17) and ***girafa/**giraffe* (41). The IPG_2 type occurred in <*anima**o*** > and < *anima**u*** > because at the end of a word, in decreasing diphthongs, the semivowel [w] can be encoded as <l>, <u>, or < o > (Scliar-Cabral, 2003). The same type was observed in <***j**irafa* > because /Ʒ/ and /i/, forming the syllable /Ʒi/, being an irregular situation, can lead to <gi> or < ji>. In <*gi**rr**afa*>, observe the type IPG_1.

Second to last on the ‘DSW’ list are the target words ***carro/**car* (18), ***galinha/**chicken* (60), and ***porque/**because* (44), which were written, each one, incorrectly by three students from GD. In the target word ***carro/**car*, the students wrote it without using the digraph <rr>: <caro>, resulting in an error of type IPG_1. For the target word ***galinha/**chicken*, the error was the exchange of the digraph <nh> for <lh> in <*gali**l**ha*>, RPG type error. There is a preponderance of the natural spelling error for the target word ***porque/**because* in three types: regular phoneme/grapheme correspondence in <*poor**g**ue*>, with the exchange of <q > for <g>; omission or insertion of letters, with the addition of grapheme in <*po**o**rgue* > and omission of the grapheme in <*poque*>; alteration in the syllabic structure in <***pro**que*>, with a change in the order of the graphemes.

Only one GD student wrote the word target ***menino/**boy* (16), replacing <e > with <i>, in <*m**i**nino*>, making the error of AS classified as IPG_2.

The target words ***casa/**house* (19) and ***escola/**school* (37) were the only ones written orthographically by all 60 students in the study, even though they represent irregular words, with the syllables /za/ in ***casa/**house*, which could be written by <sa> or < za>, and /es/ in ***escola/**school*, which could be written by <es> or < ex>, suggesting that they do not offer difficulties in terms of their notation because they are words of high frequency in the Portuguese language, both in reading and writing, from the first years of Elementary School I (Oliveira et al., 2021).

## 5 Discussion

In the last decades, researchers have developed several spelling scoring methods, which are fine-grained alternatives to the traditional correct/incorrect scoring. Those methods provide detailed information about the challenges imposed by spelling in varying writing systems (Treiman et al., 2019).

Portuguese is an orthography of intermediate depth (Seymour et al., 2003) that has several complex as well as inconsistent spelling features. Studies focusing on the acquisition of these features by Portuguese children are scarce (Mesquita et al., 2020).

A careful analysis of the mistakes made by students with learning disabilities in writing reveals a number of qualitatively distinct errors, each of which is directly related to one of the basic fundamental processes for spelling: perceptive-linguistic, metalinguistic, operative memory, and long-term memory processes, among others (Cervera-Mérida and Ygual-Fernández, 2006).

The analysis of spelling errors in the list ‘Dyslexic Sight Words – DSW’ by semiological classification provides explanations that can contribute to the formal teaching of orthographic writing in Brazilian Portuguese. From an evolutionary point of view, all categories of errors were observed in all of the Dyslexia Group school years, with the arbitrary spelling error categories irregular phoneme/grapheme correspondence type 1 and irregular phoneme/grapheme correspondence type 2 being the most frequent throughout the Dyslexia Group’s entire schooling.

The present study demonstrates that both groups, GD and GWD, made more arbitrary spelling errors than natural spelling over the school years. The justifications raised for the spelling disorders presented by the students in this research were mainly guided in two directions. The first refers to the difficulty that the written code itself brings in the face of irregularities in our language, spoken and written. The second concerns the relationship of this difficulty with failures in phonological, visual, and orthographic working memory processing in students with dyslexia.

Among the categories of natural spelling, errors due to unconventional segmentation of word were frequently observed in students from GD3, GD4, GD5, and GD6 in words frequently used in the Portuguese language: ***de repente/**suddenly*, ***por isso/**therefore*, ***em cima/**above,* and ***embaixo**/under*. An essential element of learning to read and write at school is the ability to define word boundaries. Two types of errors are possible: hyposegmentation and hypersegmentation. Hyposegmentation occurs when there is a failure to separate two or more words with a space and hypersegmentation occurs when words are split into more than one segment, that is, a blank space is imposed within a conventional word (Correa and Dockrell, 2007).

Omission of letters was also a frequent error category even in GD5 students when omitting the letter r at the end of common verbs in the Portuguese language: ***correr/**run*, ***viajar/**travel,* and ***enxergar/**see*. Among all error categories, the alteration in the syllabic structure type was only observed in GD, more specifically in GD3, when reversing the order of letters in the word. Some spelling errors in the regular phoneme/grapheme correspondence category that concern the replacement of graphemes that represent homorganic phonemes (e.g., /t/ and /d/) were not seen in GD6. When writing the word ***conseguiu/**got*, for example, students from GD3, GD4, GD5, and GD6 made more than 15 errors in the irregular phoneme/grapheme correspondence type 1—arbitrary spelling category. In the same sense, students from GD3 to GD6 made errors in the irregular phoneme/grapheme correspondence type 2 when writing the word ***assalto**/assault* in eight diverse ways.

Spelling difficulties characterize dyslexia across alphabetic writing systems; the systems themselves can vary considerably in how they encode spoken language. Thus, the type of knowledge that learners need to bring to the spelling task may vary as a function of the language they speak and write (Caravolas and Volín, 2001). The high number of inaccuracies observed in the writing of the group with dyslexia (GD) was often related to the complexity of syllabic structures and orthographic irregularity. The syllabic structures, in addition to the consonant-vowel (CV) pattern, often pose challenges for all students as they move through the alphabetic writing phase, at the beginning of literacy. The complexities and inconsistencies in an orthographic system make spelling acquisition more challenging for beginning writers (Serrano et al., 2011); the GD3 presents persistent difficulties in complex syllabic spelling, as in this case, there is a greater demand on cognitive–linguistic skills, mainly on phonological and spelling operational memories and on long term spelling memory.

In the group with dyslexia (GD) and the group without dyslexia (GWD), incorrect notations of words that should be accented were observed, with the alteration in accentuation of words type error appearing, suggesting that the teaching of accentuation is an aspect to be worked on. Furthermore, in dyslexia, the difficulty of accentuation is even greater because the correct use of accentuation is related to aspects of stress and syllabic separation.

It is opportune to emphasize that, in the teaching of the orthographic norm, the success and error when writing words should be followed by a reflection (metaorthographic skill) and monitoring of learning, both on the part of the teacher and on the part of the learner, reinforcing the knowledge of spelling patterns that will be triggered as the student is exposed to the explicit formal teaching of spelling. This point opens up new and necessary possibilities for future research.

## 6 Conclusion

The analysis of spelling errors in the list ‘Dyslexic Sight Words – DSW’ organized by semiological classification provides explanations that can contribute to the formal teaching of orthographic writing in Brazilian Portuguese. The high number of inaccuracies observed in the writing of the group with dyslexia (GD) was often related to the complexity of syllabic structures and orthographic irregularity. The syllabic structures, in addition to the consonant-vowel (CV) pattern, often pose challenges for all students as they move through the alphabetic writing phase early in literacy. In both the group with dyslexia (GD) and the group without dyslexia (GWD), incorrect notations of words that should be accented were observed with alteration in accentuation, suggesting that the teaching of accentuation is an aspect to be worked on. Furthermore, in dyslexia, the difficulty of accentuation is even greater because its correct use is related to aspects of stress and syllabic separation. The use of patterns of oral communication in writing was also observed in the spelling errors of GD.

This classification provides an understanding of the characteristics of natural spelling and arbitrary spelling, providing support for the teaching-learning of words by dyslexic students and is also relevant for the design of Portuguese language teacher training policies. An important issue to highlight is the fact that our study is a pioneering work in the Brazilian Portuguese scenario with regard to the semiological classification of spelling errors. No previous research has been conducted focusing on spelling errors in dyslexic students, conducting the semiological classification of a specific list composed of 60 words frequently written incorrectly by students with dyslexia, which allows specific interventions in the types of errors in the semiological classification presented here.

## Future research

This study aims to analyze in detail the spelling errors of the ‘Dyslexic Sight Words – DSW’ list according to the semiological classification made by Brazilian schoolchildren.

From an evolutionary point of view, all categories of errors were observed in all of the Dyslexia Group school years. An analysis of the categories of errors, by word and by school year, will be considered in future work.

## Data availability statement

The original contributions presented in the study are included in the article/supplementary material, further inquiries can be directed to the corresponding author.

## Author contributions

LC and AB contributed to the conception and design of the study, analysis and interpretation of data, writing of the article, and final review. FM and SC contributed to the design, supervision and preparation of the study, corrections, and final review. All authors contributed to the article and approved the submitted version.
